# Advancing sustainability in low-resource settings: development and validation of a sustainability tool for evidence-based interventions and programs

**DOI:** 10.3389/frhs.2025.1618400

**Published:** 2025-07-24

**Authors:** Chisom Obiezu-Umeh, Divya S. Subramaniam, Ucheoma Nwaozuru, Titilola Gbaja-biamila, Lateef Akeem Blessing, Thembekile Shato, David Oladele, Lisa R. Hirschhorn, Enbal Shacham, Hong Xian, Oliver Chukwujekwu Ezechi, Juliet Iwelunmor

**Affiliations:** ^1^ Department of Medical Social Sciences, Northwestern University Feinberg School of Medicine, Chicago, IL, United States; ^2^ Department of Health and Clinical Outcomes Research, Saint Louis University School of Medicine, Saint Louis, MO, United States; ^3^ Department of Implementation Science, Wake Forest University School of Medicine, Winston-Salem, NC, United States; ^4^ Division of Infectious Diseases, Washington University in St. Louis School of Medicine, Saint Louis, MO, United States; ^5^ Clinical Sciences Department, Nigerian Institute of Medical Research, Lagos, Nigeria; ^6^ Department of Surgery, Washington University in St. Louis School of Medicine, Saint Louis, MO, United States; ^7^ College for Public Health and Social Justice, Saint Louis University, Saint Louis, MO, United States

**Keywords:** sustainbility, determinants, measurement development, psychometric properties, implementation science outcomes

## Abstract

**Introduction:**

Despite substantial research and growing evidence on effectiveness, the longer-term benefits of proven healthcare interventions and programs have not been fully explored due to challenges sustaining such efforts. Existing sustainability measures developed in high-income countries may not reflect determinants unique to the sustainability of interventions in low- and middle-income countries (LMICs), including African countries. To address this gap, our study developed a Sustainability Tool to Assess Evidence-Based Interventions and Programs (STEPS), which provides a theory-based measure that can be used to assess multilevel determinants of sustainability from the perspective of frontline health workers, service providers, or implementation practitioners.

**Methods:**

STEPS domains and the initial scale item pool were generated based on a review of existing literature on sustainability in the African region. Two rounds of expert reviews were conducted with 12 experts from nine African countries, providing ratings and feedback on the relevancy of each item. Then, face validity was conducted among ten healthcare workers involved in implementing interventions and programs in Nigeria. Content validity metrics and consensus methods were used to remove redundancy, reducing the final scale to 31 items. Subsequently, we piloted STEPS among 256 healthcare workers in Nigeria directly involved in implementing evidence-based programs and/or interventions. Data were analyzed using exploratory factor analysis (EFA) to identify the underlying factor structure, followed by reliability analysis.

**Results:**

The EFA indicated that a four-factor 31-item structure best fits the data (Kaiser Criterion of eigenvalues >1, confirmed by scree plot, and interpretability). The four subscales are: (1) intervention characteristics (2) organizational capacity, (3) implementation context and values, and 4) socio-cultural and community context. The Cronbach's alpha for the subscales ranged from 0.83 to 0.95. Overall, STEPS demonstrated adequate content validity and excellent internal consistency for the overall scale with a Cronbach's alpha of 0.97.

**Conclusion:**

Our research findings contribute to the implementation science literature by providing future researchers or programmers a means to assess factors associated with the long-term delivery and subsequent benefits of evidence-based interventions and programs in African settings. STEPS provides a context-relevant tool for assessing sustainability in the African context and other LMICs.

## Introduction

Sustainability remains an understudied and complex issue in research and practice settings. It is one of the least understood and most vexing translational research issues of our time, largely due to unique methodological challenges (1, 2). Informed by a recent synthesis by Moore et al. 2017, sustainability is conceptualized based on five characteristics: “(a) after a defined period of time; (b) a program, clinical intervention, and/or implementation strategies continue to be delivered and/or; (c) individual behavior change (i.e., clinician, patient) is maintained; (d) the program and individual behavior change may evolve or adapt while; (e) continuing to produce benefits for individuals/systems” (3). However, despite the growing consensus on how sustainability is defined, there are no agreed-upon or uniform criteria for determining whether an intervention or program has been or will be sustained (4). This may be due to a recent shift in perspective—from viewing sustainability as an “endgame” or afterthought to recognizing it as an ongoing, dynamic process, where adaptation is critical to overcoming hurdles and responding to evolving contexts and populations (2). Given the current climate of shifting priorities, competing demands, and limited resources, particularly in low- and middle-income countries (LMICs), understanding how to sustain interventions and programs effectively is crucial to maximizing public health impact and avoiding wasted investments.

Current literature highlights a myriad of methodological challenges in assessing sustainability across diverse settings. Sustainability is often not assessed, and when measured, different metrics and observation periods have been used (5–7). In a review of 84 funded implementation research studies, only 67% of studies referenced sustainability, and none of the studies mentioned planning for sustainability from the onset of implementation (8). Comparable results were found in a review of 41 health interventions in Africa, where only 46% of the included studies reported sustainability outcomes, most of which exhibited limited quality and methodological rigor (9). More recently, Hall et al. (2023) conducted a comprehensive review of over 10,000 public health research articles and found that only 1.3% (*n* = 136) were focused on sustainability, with only two studies specifically addressing measurements (10). Additionally, many studies do not use measures that have been empirically tested for substantive or predictive validity (11). For example, sustainability is sometimes assessed by the percentage of sites that continued program activities after funding ended or by the rate of outcome improvement (1, 11). However, simply determining whether a program or its components continued does not guarantee that the program sustained benefits for the same number of beneficiaries, nor does it confirm continued effectiveness beyond the initial funding period. Given that most data collection activities conclude when grant funding stops, information about sustainability is typically beyond the purview of rigorously controlled research trials or health service research (1). As such, the underdeveloped state of sustainability measurement poses one of the most serious methodological challenges in implementation science.

Existing measures are limited in three critical ways. First, nearly all the measures were conceived and validated in high-income countries (HICs) (4–6, 12, 13). Studies have shown that implementation and sustainability determinants might manifest differently in LMICs and HICs due to variations in health system structures, resource availability, morbidity and mortality population-level profiles, and cultural and sociopolitical norms (9, 12, 14). Thus, measures may require adaptation or contextualization to fit the needs and priorities of researchers, practitioners, communities, and policymakers in LMIC settings. Second, existing measures provide little insight into the multidimensional nature of sustainability (1, 15) or whether it may be influenced by important determinants such as (a) continuation of intervention benefits (15) (b) how program activities of the original intervention fit the context where they are implemented (9, 16); (c) how to maintain dialogue with stakeholders and leaders to continually improve practices or procedures that were started as a result of program implementation; or (d) how to engage in learning or problem-solving at multiple levels to boost sustainment in practice (16, 17). Finally, existing measures are better suited for executive or management staff with higher-level organizational knowledge rather than frontline health workers or service providers, who have firsthand experience with daily operations at clinics and implementation sites. Although valuable, such tools fall short of capturing the frontline health workers or service providers assessment of what is truly happening at the ground level of implementation.

The current study does not seek to replace existing frameworks or tools for understanding sustainability determinants or outcomes. Rather, it integrates domains and constructs from locally contextualized frameworks that reflect the realities of LMICs, particularly in Africa, to provide a pragmatic tool to assess and guide the sustainability of interventions and programs in low-resource settings. Accordingly, we developed and validated the sustainability tool to assess evidence-based interventions and programs (STEPS). STEPS aims to evaluate core sustainability determinants that support the continued delivery of innovations. This article describes the conceptualization and development of STEPS and presents psychometric evidence supporting its potential use as a robust measure to advance sustainability research and practice.

## Materials and methods

**Figure 1:**
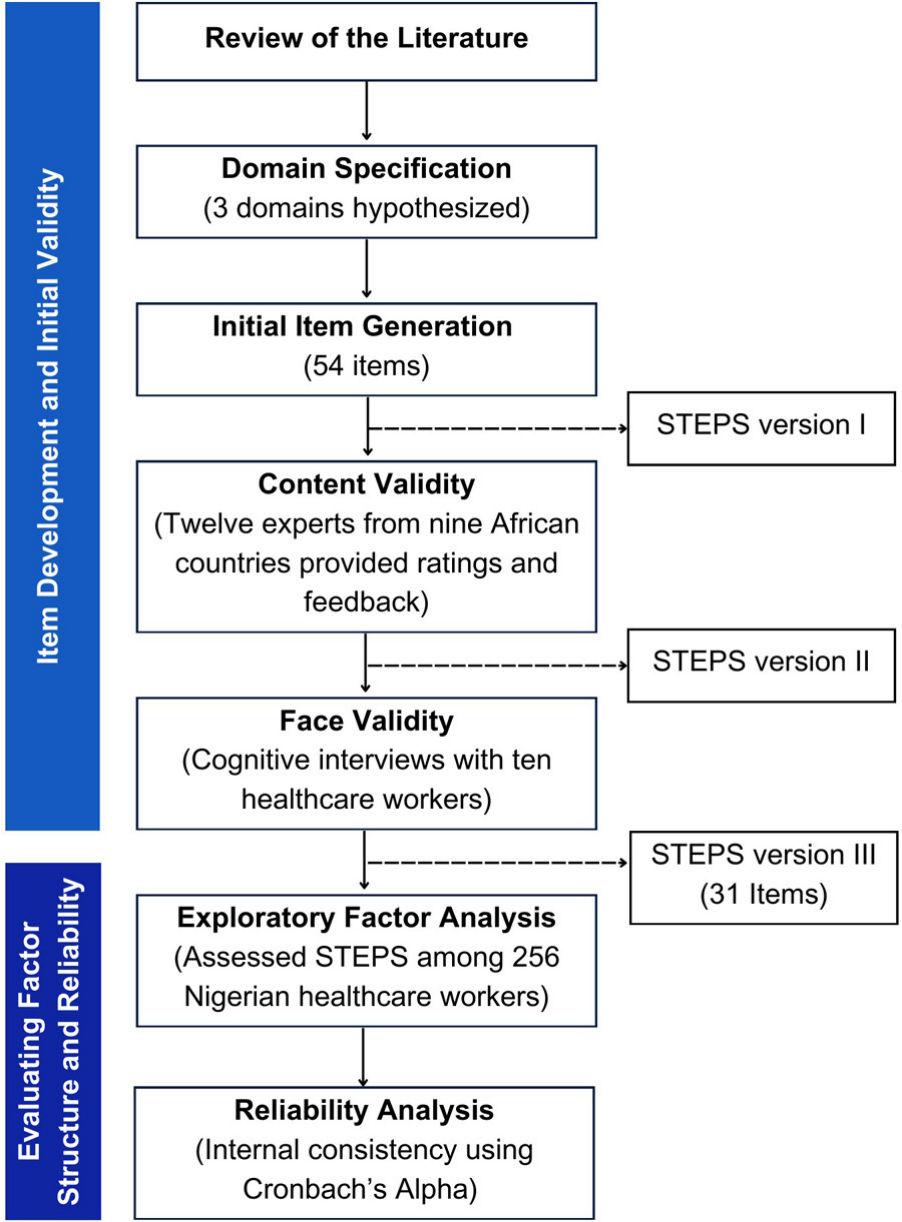
STEPS development and evaluation process.

Following the best practice guidelines described by Boateng, we used a multi-stage, mixed-methods approach to develop and evaluate STEPS from November 2022 to December 2023 (see Figure 1).

### Phase I: item development and initial validity

#### Defining dimensions and developing the initial item pool for STEPS

Guided by the Dynamic Sustainability Framework (DSF) (16), along with the conceptual framework developed from the published literature by Iwelunmor et al., (2016) (9), we delineated and operationalized the domains for STEPS (See Supplementary Table 1, Additional File 1). Scale items were then deductively created after a comprehensive review of the literature on sustainability determinants and outcomes in the context of LMIC (9, 18). These items were derived from central themes identified in the existing literature and mapped to the initial three hypothesized STEPS domains: intervention characteristics, organizational implementation context, and socio-cultural and community context (See Supplementary Table 1, Additional File 1). Following the recommendation by Clark and Watson (19) for item generation, the initial item pool was at least twice as long as the final version of the scale. A larger item pool at the initial phase would protect against poor internal consistency and ensure that the best possible item represents the underlying construct (20). A preliminary scale with 54 items (Version I), categorized under the three domains, was then created. Following the item generation process, content validity by experts and face validity by a subsample of healthcare workers were conducted.

#### Content validity

Twelve subject matter experts (SMEs) were purposively selected, following widely used recommendations (2–20 SMEs) (21–23), and their extensive experience in leading and implementing healthcare interventions or programs in Africa. All SMEs had implemented large-scale programs in at least one African country, including Zambia, Nigeria, Botswana, Rwanda, Malawi, Tanzania, Kenya, and South Africa. SMEs were invited via email with a formal cover letter detailing the study's purpose, domain definitions, item rating instructions, and a Qualtrics survey link with open-ended questions for additional feedback. Experts independently rated each item's relevance (1 = not relevant to 4 = highly relevant) and provided qualitative feedback on item inclusion, rewording, deletion, and response options. Quantitative data were imported into Microsoft Excel, and the content validity index (CVI) was calculated (See Supplementary Table 2, Additional File 1). The Item-Level CVI (I-CVI) was determined as the proportion of experts rating an item as 3 (quite relevant) or 4 (highly relevant) out of the total number of experts. Items with I-CVI <0.70 were considered for deletion, while those scoring 0.70–0.79 were reviewed for modification based on expert feedback. The Scale-Level CVI (S-CVI/Ave) was 0.83, exceeding the 0.80 threshold for acceptable content validity (22, 24). In light of these findings, items were refined, and STEP Version II was developed.

#### Face validity

To understand how target group respondents perceived and understood STEPS version II, we conducted cognitive interviewing (CI) using the “think-aloud” technique with ten healthcare workers in Nigeria. Participants (aged 18 and older) had experience implementing or leading healthcare interventions and included physicians (*n* = 3), nurses (*n* = 1), community health workers (*n* = 5), and pharmacists (*n* = 1). Interviewees were asked to verbalize their thought process while responding to each scale item, guided by MacDermid J's taxonomy to evaluate clarity/comprehension, relevance, and perspective modifiers (25). Trained study team members with extensive qualitative research experience conducted in-person interviews in English lasting 30–45 minutes. Using rapid qualitative analysis (26), interview responses, and field notes were analyzed to refine the scale, resulting in 31 final items (version III).

### Phase II: psychometric evaluation

#### Participants, setting, and procedure

To assess the factor structure (i.e., how many underlying factors might be present and their relationship to each other) and reliability, implementers including health service providers, involved in implementing HIV-focused programs and interventions in Nigeria for at least 12 months or those recently completing such programs were invited via email to complete the STEPS measure (Version III) through the Qualtrics web-based platform. We focused on HIV programs as a starting point because they represent the first large-scale continuity care programs implemented in many LMICs and share similar prevention and management elements with noncommunicable diseases and other infectious diseases in this region (27). Principal investigators of active and completed HIV-focused programs in Nigeria were identified through implementation research networks and asked to connect our evaluation team with healthcare providers involved in these programs. Providers were invited to participate, and additional referrals were obtained. Following recommendations for scale development and factor analysis, we aimed for a sample size of 300 respondents (to account for non-responses) to pilot test STEPS (28, 29). Participants accessed the secure Qualtrics platform, provided informed consent, completed a set of eligibility questions, and completed the STEPS survey. The survey platform remained open for two months, with email reminders sent after the initial invitation. Respondents rated the items based on their specific program or intervention using a 5-point Likert scale (“1 = strongly disagree”, “2 = disagree”, “3 = neither agree nor disagree”, “4 = agree”, “5 = strongly agree’), with an additional option for “Not applicable to my role or intervention/program at this time.” At the end of the survey, participants provided demographic information, including age, sex, education level, position, and practice/program characteristics.

#### Data analysis

Deidentified data were extracted from Qualtrics and analyzed using IBM Statistical Package for the Social Sciences (SPSS), version 28. Item responses and response patterns were first examined for missing data and poorly answered items for potential exclusion. Responses with over 20% missing data were excluded. Frequency tables were generated to describe the socio-demographics of participants in the study. Univariate skewness and kurtosis were examined to assess whether the data was normally distributed. Skewness, with an absolute value of less than 3, and Kurtosis, with an absolute value of less than 10, are the suggested ranges (30). Inter-item correlations were computed to examine the homogeneity of the scale (minimally acceptable range of r ≥ 30) and to identify highly correlated items (r > 0.90), potentially indicating multicollinearity (31).

Next, The Kaiser-Meyer-Olkin (KMO) (32) and Bartlett's test of sphericity (33) were used to determine the appropriateness of the data for factor analysis. The data was considered adequate if the KMO was >0.60 and Bartlett's test was statistically significant (*p* < 0.001). Several approaches were used to determine the number of factors to retain, including Kaiser's criterion (eigenvalues >1) (34), cumulative percent of variance explained, and the scree plot (identifying the point where the curve flattens) (35). Factor extraction was conducted using principal axis factoring (PAF) with oblique rotation (Promax), which is preferred for new measure development (36, 37). Oblique rotation was chosen based on the assumption that factors were correlated (37). Factor solutions were subsequently examined, and items with loadings below 0.30 or factors with fewer than three items were not retained. Given that no prior hypotheses had been established regarding the underlying dimensions, EFA was deemed more appropriate (31).

#### Internal consistency

Internal consistency was determined using Cronbach's alpha for the total scale and subscales identified by the EFA. Cronbach's alpha values of 0.80 or higher are considered excellent internal reliability (31).

#### STEPS scoring

Sums and mean scores were computed for the overall scale and subscales using unweighted (mean or sum scores) items. The scores associated with each factor served as the composite scale score. Potential scores for the scale items range from 1 to 5. Higher scores (typically within 4–5) may be interpreted as higher capacity for sustainability in each area.

## Results

### Demographic characteristics of study population in phase II

Of the 312 responses received, 56 surveys were excluded due to over 20% missing data, resulting in 256 responses for factor analysis (82.0% response rate). Table 1 presents the sociodemographic characteristics of the final sample. Most respondents were aged 25–34 years (36.3%), female (57.1%), and held a bachelor's degree or higher (59.4%). Regarding professions, 34.8% were community health workers, 30.9% were counselors or social workers, 12.5% were medical doctors, 6.3% were nurses/midwives, 2.0% were pharmacists, and 13.7% were other allied health professionals. Most worked in tertiary hospitals (31.3%) or primary healthcare centers (27.7%), and over a third reported that the HIV-based program or intervention had been in implementation for 10 years or more (38.3%).

**Table 1:** Demographic characteristics of study population in phase II (*N* = 256).

Measures	Overall
	*n* (%)
Age, years	
18–24	61 (23.8)
25–34	93 (36.3)
35–44	53 (20.7)
45–54	36 (14.1)
55–64	13 (5.1)
Sex	
Female	145 (57.1)
Male	109 (42.9)
Missing	2
Educational attainment	
Less than bachelors’ degree^a^	102 (40.6)
Bachelors’ degree or higher	149 (59.4)
Missing	5
Profession	
Medical doctor	32 (12.5)
Nursing/midwifery	16 (6.3)
Community health worker/peer health navigator	89 (34.8)
Pharmacist	5 (2.0)
Counselor/social worker	79 (30.9)
Other ^b^	35 (13.7)
Type of facility currently working in	
Primary (PHC)	71 (27.7)
Secondary (general/district hospital)	54 (21.1)
Tertiary/Referral center (teaching or specialist hospital)	80 (31.3)
Community (health post or clinics)	23 (9.0)
Other ^c^	28 (10.9)
Health facility type	
Private	45 (17.6)
Public	211 (82.4)
Duration of program/intervention implementation, years	
1–2	44 (17.2)
3–5	47 (18.2)
6–10	61 (23.8)
>10	98 (38.3)
Do not know	6 (2.3)

PHC, Primary Healthcare Center.

^a^ Includes primary education, secondary education, some college, technical college, diploma certificate.

^b^ Other professions include health facility staff such as lab technicians, medical student trainees or other allied health professionals.

^c^ Other facilities include research centers/institutes, and non-governmental organizations.

### Descriptive statistics

All items demonstrated univariate normality, with item skewness ranging from −1.91 to −0.24. The Kurtosis values ranged from −1.09 to 5.55. The values for both skewness (absolute values less than 3) and Kurtosis (absolute values less than 10) were within the suggested ranges (30); thus, the results from the descriptive statistics indicated it was appropriate to conduct further analysis. Bivariate correlations were then examined to check for correlations <0.30 or >0.90. Most of the items were significant and moderate in magnitude. The item correlations ranged from −0.04 to 0.81. There were no pairs of items that had correlations higher than 0.90, which indicated that there were no cases of multicollinearity.

### Sampling adequacy

The Kaiser-Meier-Olkin (KMO) measure of adequacy was 0.959, and Bartlett's test of Sphericity was statistically significant (*χ*^2^ = 6,237.597, *df* = 465, *p* < 0.001). Given that the KMO was higher than the suggested value [KMO > 0.60 (38)] with a significant Bartlett's test of sphericity, this indicated that the sample (*n* = 256) was adequate for exploratory factor analysis.

### Exploratory factor analysis

**Figure 2:**
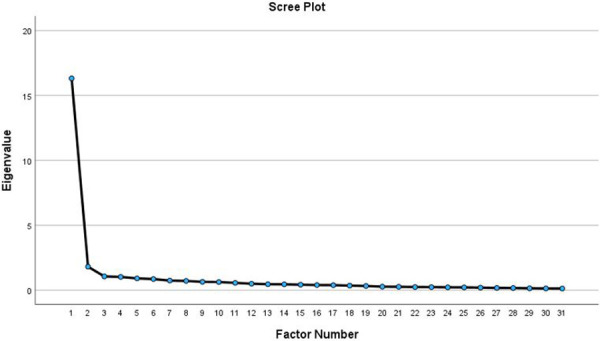
The scree plot graph.

Based on Kaiser's criteria (eigenvalues >1), the four-factor solution was suggested, which accounts for 65.19% of the total variance. The eigenvalues of factors 1, 2, 3, and 4 are 16.31, 1.81, 1.06, and 1.02, respectively, as presented in Table 2. The scree plot in Figure 2 showed a break at the third eigenvalue, where the data began to flatten, thereby suggesting a three-factor solution.

**Table 2:** Eigenvalues for the kaiser criterion.

Factor	Initial eigenvalues			Extraction sums of squared loadings		
	Total	% of variance	Cumulative %	Total	% of variance	Cumulative %
1	16.314	52.624	52.624	15.935	51.402	51.402
2	1.813	5.847	58.471	1.372	4.427	55.830
3	1.060	3.420	61.891	0.671	2.165	57.995
4	1.023	3.301	65.192	0.668	2.155	60.150

A three-factor structure of the sustainability measure was initially expected based on prior research and conceptual framework. However, substantial cross-loadings made it difficult to assign meaningful factor names due to the broad range of item content within each factor, masking critical conceptual distinctions. Though not presented here, this solution accounted for 61.89% of the total variance and was deemed a poor fit.

A four-factor solution, explaining 65.19% of the variance, was further examined. Items with factor loadings above 0.30 were considered adequate candidates for inclusion (Table 3). Factor 1 (12 items) assessed organizational aspects related to workforce capacity, alignment with workflow, sustainability planning, leadership support, funding, resources, management, and monitoring. Factor 2 (9 items) captured the organizational implementation context and value domain, including culture, climate, beliefs, and perceived benefits. Factor 3 (7 items) reflected socio-cultural and community context, covering stakeholder involvement, resources, knowledge exchange, and collaboration. Factor 4 (3 items) assessed intervention fit, adaptability, and affordability for the target population.

**Table 3:** EFA factor loadings of four-factor solution.

Item		Factor loading			
		1	2	3	4
Factor 1: Organizational capacity					
1	The organization has a system in place to ensure EBI delivery continues if the key person (leads/coordinates) leaves	**0**.**870**	−0.278	0.141	0.062
2	The organization has a key person (leads/coordinates) who drives the EBI forward	**0**.**724**	0.007	−0.003	0.187
3	Plans/guidelines are in place from the start to ensure the ongoing delivery of the EBI	**0**.**684**	0.161	0.054	−0.010
4	The organization is willing to seek other sources of funding to support the ongoing delivery of the EBI	**0**.**683**	0.026	−0.058	−0.026
5	The organization's leadership team supports EBI delivery	**0**.**676**	0.213	−0.010	−0.010
6	The organization provides follow-up care/support/treatment options for the target population	**0**.**656**	0.138	−0.043	0.047
7	The organization has sufficiently trained staff who are able to continue the EBI delivery	**0**.**596**	0.026	0.266	0.013
8	The organization is committed to the continued delivery of the EBI	**0**.**534**	0.401	−0.030	−0.060
9	Procedures are in place to monitor whether EBI is being delivered as intended	**0**.**495**	0.216	0.050	0.102
10	A system exists to communicate the expectations and changes related to the EBI delivery	**0**.**487**	0.291	0.176	−0.061
11	The EBI aligns well with the current services provided in the organization	**0**.**420**	0.329	0.016	0.128
12	The organization commits sufficient resources to support the EBI delivery	**0**.**373**	0.305	0.116	−0.008
Factor 2: Organizational implementation context and value(s)					
13	Staff members have a clear understanding of how to deliver the EBI	0.058	**0**.**809**	−0.123	0.071
14	The EBI is in line with the priorities of the organization/ community where it is carried out	−0.178	**0**.**781**	0.293	−0.107
15	Staff members understand the benefits of using the EBI for the target population	0.160	**0**.**673**	−0.086	0.069
16	Staff members feel confident delivering the EBI	0.439	**0**.**566**	−0.090	−0.041
17	The staff members find the delivery of the EBI to be straightforward	−0.067	**0**.**544**	0.009	0.347
18	The EBI aligns well with the goals of the organization	0.343	**0**.**525**	−0.113	0.022
19	The EBI aligns with the culture and values of the target population	0.071	**0**.**492**	0.209	−0.067
20	The organization recognizes staff members who use the EBI in their routine activities	0.123	**0**.**490**	0.113	0.035
21	Staff members spend adequate time on the delivery of the EBI	0.363	**0**.**472**	−0.035	0.050
Factor 3: Socio-cultural and community context					
22	Community leaders play an active role in the delivery of the EBI	−0.084	−0.131	**0**.**831**	0.042
23	The community has access to knowledge and information about the EBI	0.070	−0.007	**0**.**774**	−0.001
24	The EBI encourages mutual learning with community partners/organizations	0.108	0.063	**0**.**645**	−0.099
25	The local government supports the delivery of the EBI	−0.020	0.007	**0**.**632**	0.136
26	The [target population] is actively involved in influencing how the EBI is carried out	−0.020	0.125	**0**.**567**	0.095
27	The EBI builds on the resources and strengths of the community	0.197	0.089	**0**.**417**	−0.107
28	Diverse network of partners (government, private, non-profit, or community) are actively involved in the delivery of the EBI	0.184	0.241	**0**.**361**	0.017
Factor 4: Intervention characteristics					
29	The EBI delivery can be adapted (to make more suitable) to address changes in the community/organization	0.274	−0.166	−0.029	**0**.**782**
30	The EBI meets the needs of the target population in the community	0.122	0.133	0.011	**0**.**600**
31	The target recipients find the EBI to be affordable	−0.199	0.368	0.110	**0**.**594**

*N* = 256. Extraction method: principal axis factoring with an oblique (Promax) rotation. A pattern matrix was used to assess factor loadings. Factor loadings above 0.30 are in bold/underlined. EBI, evidence-based intervention/program/practice.

Correlations among the four factors were examined (Table 4). Results indicated that Factors 1 and 2 were closely related (r = 0.79), suggesting they tap into a similar construct, though not enough to indicate redundancy. Other factors showed moderate correlations (ranging from 0.47 to 0.67).

**Table 4:** Four-factor solution inter-factor correlation matrix.

Factor	1	2	3	4
1	1.000			
2	0.793	1.000		
3	0.655	0.641	1.000	
4	0.666	0.646	0.469	1.000

### Reliability analysis

Cronbach's alphas were calculated to assess the internal consistency of the subscales and the full 31-item scale (Table 5). The overall Cronbach's alpha was 0.968, indicating excellent reliability. Subscale reliability was also strong: Factor 1 (Organizational Capacity) had an alpha of 0.95, Factor 2 (Organizational Implementation Context and Values) was 0.92, Factor 3 (Socio-Cultural and Community Context) was 0.86, and Factor 4 (Intervention Characteristics) was 0.83.

**Table 5:** STEPS subscale and overall means, standard deviations, and cronbach's alpha.

STEPS domain	Number of items	M	SD	Cronbach's Alpha
Factor 1: Organizational capacity	12	4.03	0.763	0.950
Factor 2: Organizational implementation context and Values	9	3.98	0.707	0.922
Factor 3: Socio-cultural and community context	7	3.84	0.659	0.864
Factor 4: Intervention characteristics	3	3.94	1.031	0.833
Overall STEPS score	31	3.96	0.127	0.968

The overall score represents the average responses on the 1–5 Likert scale for all 31 items. Each subscale score represents the average responses of the items in that scale. M, mean; SD, standard deviation.

### Scale scoring

Subscale and overall STEPS scores were calculated (Table 5), with potential item scores ranging from 1 to 5. The mean STEPS score was 3.96 (SD = 0.127). Among the domains, Organizational Capacity had the highest mean (M = 4.03, SD = 0.763), followed by Organizational Implementation Context and Values (M = 3.98, SD = 0.707) and Intervention Characteristics (M = 3.94, SD = 1.031), which had similar scores. The Socio-Cultural and Community Context domain had the lowest mean (M = 3.84, SD = 0.549). Higher scores (typically 4–5) indicate greater capacity for sustainability in each domain.

## Discussion

The current study developed and evaluated the 31-item sustainability measure, which provided a theory-based measure that can be used to assess multilevel determinants of sustainability from the perspective of frontline health workers who are on the ground, directly delivering an intervention or program. This study also offers foundational insights suggesting that intervention characteristics, organizational capacity, implementation context, and socio-cultural and community context are key constructs that warrant consideration when evaluating sustainability.

Importantly, STEPS was not developed in isolation but rather builds on already existing, well-known implementation frameworks, offering a much-needed, contextually tailored measure for African settings and other LMICs. The approach used for developing STEPS and the result of this process supports Glasgow and Riley's (2013) [164] call for more pragmatic measures in implementation science that are psychometrically sound and can be used in real-world contexts. Additionally, it adheres to recommendations by Martinez et al. (2014) for instrumentation in implementation science, which emphasize the use of frameworks, theories, and models, the development of “home-grown” measures, and the selection of appropriate evaluation approaches, all while ensuring that the measurements remain practical for use (39).

Following these principles, the development and testing of STEPS involved an iterative process that engaged diverse stakeholders to evaluate the representativeness and relevance of the items, as well as the usability of the measure by implementers including frontline health workers and service providers. As part of the Phase II evaluation, participants had been involved in program implementation for at least 12 months, consistent with another validation study assessing a sustainment outcome measure (40). The data encompassed programs and interventions with implementation durations ranging from 1 to over 10 years, representing a continuum from not yet sustained to sustained.

Although a three-factor solution was initially hypothesized based on the Dynamic Sustainability Framework, along with the conceptual framework developed from the published literature by Iwelunmor et al., (2016) (9), the EFA revealed a four-factor structure, following best practices for factor retention criteria, including eigenvalues above 1, cumulative percent of variance explained, and interpretability. Two factors (intervention characteristics and socio-cultural and community context) were identical to the hypothesized domain. The two other factors represented the hypothesized organizational implementation context domain split into two factors. Content analysis of the items loaded in the two emerging factors (organizational capacity and organizational implementation context and value) revealed key conceptual differences between both domains.

The uniqueness of STEPS lies in its inclusion of the socio-cultural and community context domain, which has not been a part of other tools (13). It captures critical outer-setting factors— including values, multisectoral partnerships, community engagement, and assets—that shape sustainability at the community level, particularly in African settings. Literature highlights the vital role of communities in improving health outcomes, including prevention, treatment, service delivery, and implementation success (41–44). To achieve equitable public health outcomes, community involvement must go beyond tokenism, as communities play a crucial role in sustaining initiatives and extending their impact beyond healthcare facilities (43). In under-resourced health systems, such as those in many African countries, leveraging community capacities enhances health service delivery, and multisectoral partnerships between implementing organizations and communities can significantly strengthen sustainability (45). Studies have shown that establishing multisectoral partnerships between the organization responsible for the intervention and communities can significantly strengthen the sustainability capacity of the intervention (41, 46). Additionally, aligning community needs and priorities with intervention goals is a key predictor of sustainability, as studies show that engagement is most effective when interventions align with the community's capacity and assets (47, 48).

Regarding reliability, STEPS also demonstrated excellent internal consistency for the overall scale (Cronbach's alpha = 0.968) as well as for all four subscales (Cronbach's alpha ranged from 0.83 to 0.95). Additionally, the mean scores of the overall scale and each subscale were computed to establish a scoring system for STEPS. The decision to use mean scores serves a twofold purpose within the tool. Firstly, it enables users to compare and evaluate individual subscale scores and the overall score, thus enhancing the ease of use and interpretation of the scores. Secondly, STEPS can be used by implementation practitioners, evaluators, or researchers to reflect on the implementation processes and identify areas that may either impede or facilitate sustainability capacity, as indicated by the mean scores of the subscales (i.e., higher mean scores reflect greater capacity for sustainability in a given area).

A comprehensive assessment of sustainability determinants can significantly enhance implementation efforts. Conducting such assessments throughout the implementation lifecycle can (a) inform action-oriented planning to strengthen the intervention or program's capacity for sustainability, (b) guide internal discussions between implementation participants and the systems where the intervention/program is being implemented, (c) serve as a basis for seeking additional, in-depth information through qualitative inquiries, (d) help identify and prioritize areas in need of improvement as well as areas of strengths and, (e) provide insights for potential adaptations to the intervention or program delivery in inherently dynamic settings.

### Limitations and future directions

There are several limitations worth noting. First, the study relied on self-reported data from healthcare workers involved in implementation, which may be subject to social desirability bias. Second, the study sample in phase II evaluation consisted of frontline healthcare workers and service providers engaged in HIV intervention or programs in Nigeria, which may not be fully representative of healthcare workers across Africa and other LMICs. The decision to commence with HIV intervention and programs as a starting point is rooted in the fact that HIV programs represent the foremost large-scale chronic care initiatives in Africa (49). Over the years, significant investments have been made to establish prevention and treatment infrastructure and enhance human capacity. Research suggests that HIV programs share core elements with the management of non-communicable diseases, such as promoting good health-seeking behaviors, ensuring long-term adherence to treatment regimens, and routinely monitoring treatment outcomes (50). Given these similarities, models, and tools developed for HIV program implementation could potentially be adapted for other disease areas (50). Nonetheless, STEPS was designed as a flexible repository of items that various communities of interest, including researchers and practitioners, can readily utilize. Individuals and teams employing STEPS for assessing distinct categories of interventions and programs may need to make minimal adaptations to ensure appropriate contextualization.

While STEPS shows promise, further psychometric assessment is necessary. Future research and evaluation efforts are planned to assess predictive validity in a large-scale implementation trial, examining the association between STEPS scores and hypothesized sustainability outcomes.

## Conclusion

We developed a promising tool to address a critical gap in sustainability research by creating a measure that captures the multilevel nature of sustainability determinants and can be applied across clinical, public health, and community settings in the African context. Existing measures developed in high-income countries may not fully capture the unique determinants influencing the sustainability of interventions and programs in Africa and other LMICs. Differences in health systems, resource availability, competing priorities, socio-political norms, and population-level morbidity and mortality profiles necessitate a contextually relevant measure that reflects these realities.

## Data Availability

The original contributions presented in the study are included in the article/Supplementary Material, further inquiries can be directed to the corresponding author.
