# Supplementary material for: Advancing sustainability in low-resource settings: development and validation of a sustainability tool for evidence-based interventions and programs
Source: Front Health Serv. 2025 Jul 24;5:1618400. doi: 10.3389/frhs.2025.1618400 (PMC12328286; doi:10.3389/frhs.2025.1618400)
Supplement: Supplementary file 1 [file Table1.docx]

**Additional file 1**

| Supplemental Table 1. Domains and Sub-domains of STEPS version I, phase I. | |
| --- | --- |
| Domains | **Sub-Domains** |
| Intervention characteristics | Adaptability of the EBI delivery |
|  | Fit with the target population |
|  | Cost of the EBI for the target population |
|  | Complexity of the EBI delivery |
| Organizational implementation context | Benefit and Value of EBI delivery (attitude, motivation & behavior) |
|  | Organizational culture/support towards EBI delivery |
|  | Funding/resources support towards EBI delivery |
|  | Program champion & workforce to support EBI delivery |
|  | Leadership support for EBI delivery |
|  | Early sustainability planning |
|  | Assessment/learning |
|  | Competing priorities |
| Socio-cultural and Community Context | Fit with culture, norms & values |
|  | Community ownership |
|  | Dialogue |
|  | Coalition/collaboration/stakeholder engagement |
|  | Community assets |

Abbreviations: EBI, Evidence-based intervention/program/practice

| Supplemental Table 2. Content Validity Metrics for the Sustainability Measure (n=10 Subject matter experts) | |
| --- | --- |
| Content validity metrics | **Sustainability measure (54 items)** |
| Universal Agreement | 14 |
| Number of items with I-CVI ≥ 0.70 | 47 (87.0%) |
| Number of items with I-CVI < 0.70 | 7 (13.0%) |
| Minimum – Maximum I-CVI | 0.50 – 1.00 |
| S-CVI/Ave | 0.83 |
| Abbreviation: I-CVI – Item-level content validity index; S-CVI/Ave – Average scale-level content validity index | |
